# Energetic basis of hydrogen bond formation in aqueous solution

**DOI:** 10.1007/s00249-022-01611-2

**Published:** 2022-08-12

**Authors:** Colyn Crane-Robinson, Peter Privalov

**Affiliations:** 1 Biophysics Laboratories, School of Biology, University of Portsmouth, Portsmouth, PO1 2DT UK; 2 Department of Biology, Johns Hopkins University, Baltimore, MD 21218 USA

**Keywords:** Hydrogen bonding, Van der Waals interactions, Ionic links, α-helix, DNA double helix, Base pairs

## Abstract

The thermodynamic forces driving the formation of H-bonds in macromolecules have long been the subject of speculation, theory and experiment. Comparison of the energetic parameters of AT and GC base pairs in DNA duplexes has recently led to the realisation that formation of a ‘naked’ hydrogen bond, i.e. without other accompanying Van der Waals close contacts, is a non-enthalpic process driven by the entropy increase resulting from release of tightly bound water molecules from the component polar groups. This unexpected conclusion finds a parallel in the formation of ionic bonds, for example between the amino groups of DNA binding proteins and the oxygens of DNA phosphate groups that are also non-enthalpic and entropy driven. The thermodynamic correspondence between these two types of polar non-covalent bonding implies that the non-enthalpic nature of base pairing in DNA is not particular to that specific structural circumstance.

## Introduction

Noting that the melting temperature of the DNA duplex increased with rising GC content led immediately to the speculation that the third H-bond in GC pairs results in an extra enthalpy of base pairing (Marmur and Doty 1962). H-bond formation appears non-enthalpic (Privalov and Crane-Robinson 2020) so what experimental evidence is there for exclusively entropic H-bond formation? The formation/denaturation of an isolated 29 residue α-helix that denatures in a cooperative 2-state transition was studied by Taylor et al. (1999). At the *T*_m_ of 30 °C the enthalpy of melting was ∆H = 2.7 kJ/mol-residue and the corresponding entropy ∆S = 84 J/K mol-residue. Melting was accompanied by a small increase in the heat capacity, interpreted as resulting from the breaking and hydration of non-polar interactions within the helix, dominating the negative contribution from the corresponding hydration of polar atoms, in particular those of the peptide groups. Although such thermodynamic parameters are broadly characteristic of protein denaturation, the authors were faced with interpreting the measured enthalpy and concluded that it could be assigned entirely to the hydrogen bonds and not to the breaking of non-polar van der Waals contacts. As there was no a-priori reason for this assignment of the enthalpy the conclusion remained questionable.

## Discussion

The importance of internal interactions in nucleic acid structures was illustrated by calorimetric studies of a single stranded RNA heptamer poly(A)_7_ by Breslauer and Sturtevant (1977) for which the observed enthalpy of 12 kJ/mole adenine was all attributed to base stacking, there being no obvious basis for H-bonding. The opportunity to measure the thermodynamic parameters for the formation of an individual H-bond—without the complication of additional contributions from other interactions—presented itself in calorimetric studies of the DNA duplex. A GC base pair differs from an AT pair only in its additional H-bond if stacking interactions can be taken as independent of sequence in the well-defined duplex structure of B-form DNA, so the difference in the enthalpy/entropy of their formation provides a measure of the contributions from the single extra H-bond in the GC pair. DSC experiments with short duplexes of variable sequence were therefore used (Vaitiekunas et al. 2015) to determine the thermodynamic characteristics of forming the two individual base pairs and the table gives the resulting values at 25 °C.

**Table:** 

Base pair	∆H^coop^ (kJ/mol-bp)	∆S^coop^ (J/K mol-bp)	∆G^coop^ (kJ/mol-bp)	∆Cp (kJ/K mol-bp)
CG	− 19.0	− 36.2	− 8.2	− 0.13
AT	− 28.0	− 73.5	− 6.1	− 0.13

Contributions of CG and AT base pairs to the enthalpy, entropy, Gibbs free energy and heat capacity increment on DNA formation (strand association) at 25 °C. See Privalov and Crane-Robinson (2020)

Crystal structures and other data (e.g. Drew and Dickerson 1981; Narayana and Weiss 2009; Liepinsh et al. 1992) indicate that AT pairs are associated with ordered ‘ice-like’ water in the narrowed minor groove. This implies that the enthalpy/entropy of the formation/loss of this water must be deducted from the observed parameter to obtain the intrinsic properties of the AT pair. If such water has the thermodynamic properties of ice formation (∆H = − 6 kJ/mol, ∆S = − 22 J/K mol) and about 1.5 mol of ‘ice’ are bound per AT pair (as suggested from crystal structures) then its subtraction from the observed value of − 28 kJ/mol-bp yields an enthalpy of − 19 kJ/mol-bp and an entropy of − 40.5 J/K mol-bp for an AT pair. The intrinsic enthalpy of forming an AT pair therefore appears to be of the same magnitude as for a GC pair, meaning that the formation of H-bonds does not involve any enthalpy and the 19 kJ/mol-bp released is totally derived from other close contacts, presumably largely from van der Waals stacking interactions. It is therefore the entropy difference of about 4 J/K mol-bp that gives the advantage to H-bond formation. The overall entropy of forming the base pairs in a duplex is, of course, largely conformational and strongly negative but for the GC pair is more positive than for AT pairs by 4 J/K mol-bp, a difference presumably deriving from the release of strongly bound water molecules from the extra H-bond of the CG pair on forming the duplex.

It is a very striking and most unexpected result to find that the formation of H-bonds has no enthalpic content. Such an absolute conclusion carries some uncertainty, both from the point of view of experimental error and the contribution from ordered water bound to AT pairs. Nevertheless, the intrinsic enthalpies of the CG and AT pairs are clearly very similar and this equivalence cannot possibly be accounted for if the formation of H-bonds was strongly enthalpic, as previously assumed for the α-helix.

However, there is supportive evidence for this conclusion from a different direction: the establishment of ionic links between DNA phosphates and the amino groups of arginine and lysine side chains in DNA binding proteins (DBPs), a polar interaction similar to H-bond formation. Addition of salt (competing cations) reduces the affinity of DBP/DNA interactions considerably but has no effect upon the binding enthalpy and the lowered binding constant with increasing ionic strength is largely the result of a reduction in the entropy gain coming from cation and water release when a DBP binds to DNA.
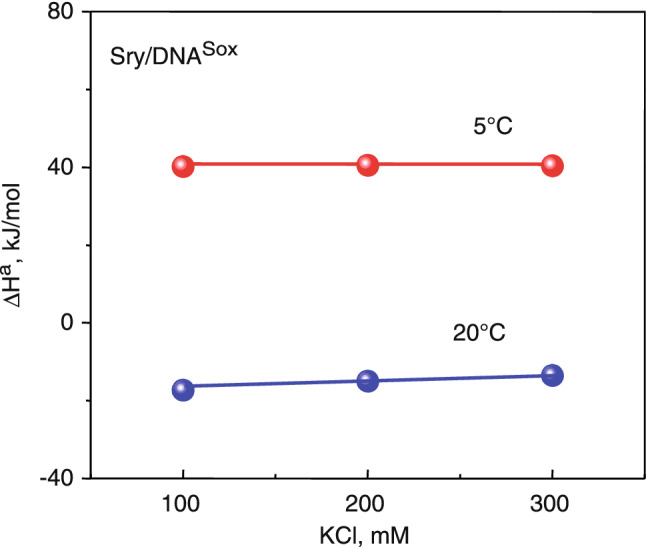


A good example to illustrate the parallel between the formation of ionic links and H-bonds comes from the complex between the HMG box of SRY protein, the product of the male sex-determining gene on the mouse Y-chromosome (for details see Dragan et al. 2004). At 20 °C and in 100 mM KCl this strong interaction is characterized by an association constant *K*^a^ of ~ 2 × 10^12^ which corresponds to a Gibbs energy of 69 kJ/mol. Of this, 48 kJ/mol (70%) comes from the 12 ionic contacts between K/R side chain amino groups and DNA phosphates. K^a^ drops to ~ 6 × 10^9^ in 200 mM KCl and to ~ 2 × 10^8^ in 300 mM KCl. The accompanying figure shows that despite reduction of the affinity by 4 orders of magnitude, ITC measurement of the enthalpy of binding at 20 °C (after correction for a small level of SRY refolding) shows no variation of ∆H with ionic strength. At 5 °C the enthalpy of binding is of very different magnitude and opposite sign (the heat capacity increment, ∆Cp, is substantial, about − 3 kJ/K mol) but is likewise independent of the state of the ionic interactions (Privalov et al. 2011). Formation of these multiple ionic links is clearly non-enthalpic and their strength comes entirely from the positive entropy generated by release of tightly bound cations from the DNA when the DBP binds.

The occurrence of such ionic bonds in DNA/DBP interactions has been discussed in detail (Yu et al. 2020) and the majority classed as CIPs (contact ion pairs) in which a hydrogen bond forms directly between the NH of a basic side chain (K/R) and an oxygen of the phosphate group. Such close ionic contacts formed between fully charged hetero-atoms separated by about 3.0 Å (the normal H-bond length) might be regarded as H-bonds, so it is unsurprising that their thermodynamic characteristic of zero enthalpy is mirrored in the hydrogen bonds formed between base pairs in DNA. The close parallel between the well-established energetics of ionic links and those proposed for H-bonding gives strong reassurance to the proposal that hydrogen bond formation in aqueous solution is a non-enthalpic process, driven by the entropy gain from release of tightly bound water molecules from the polar atoms that make up the H-bond.

## References

[CR1] Breslauer KJ, Sturtevant JM (1977). A calorimetric investigation of single stranded base stacking in the ribo-oligonucleotide A_7_. Biophys Chem.

[CR2] Dragan AI, Read CM, Makeyeva EN, Milgotina EI, Churchill ME, Crane-Robinson C, Privalov PL (2004). DNA binding and bending by HMG boxes: energetic determinants of specificity. J Mol Biol.

[CR3] Drew HR, Dickerson RE (1981). Structure of a B-DNA dodecamer. III. Geometry of hydration. J Mol Biol.

[CR4] Liepinsh E, Otting G, Wüthrich K (1992). NMR observation of individual molecules of hydration water bound to DNA duplexes: direct evidence for a spine of hydration water present in aqueous solution. Nucleic Acids Res.

[CR5] Marmur J, Doty P (1962). Determination of the base composition of deoxyribonucleic acid from its thermal melting temperature. J Mol Biol.

[CR6] Narayana N, Weiss MA (2009). Crystallographic analysis of a sex specific enhancer element: sequence-dependent DNA structure, hydration, and dynamics. J Mol Biol.

[CR7] Privalov PL, Crane-Robinson C (2020). Forces maintaining the DNA double helix. Eur Biophys J.

[CR8] Privalov PL, Dragan AI, Crane-Robinson C (2011). Interpreting protein/DNA interactions: distinguishing specific from non-specific and electrostatic from nonelectrostatic components. Nucleic Acids Res.

[CR9] Taylor JW, Greenfield NJ, Wu B, Privalov PL (1999). Calorimetric study of the folding-unfolding of an α-helix with covalently closed N- and C-terminal loops. J Mol Biol.

[CR10] Vaitiekunas P, Crane-Robinson C, Privalov PL (2015). The energetic basis of the DNA double helix: a combined microcalorimetric approach. Nucleic Acids Res.

[CR11] Yu B, Pettitt BM, Iwahara J (2020). Dynamics of ionic interactions at protein-nucleic acid interfaces. Acc Chem Res.

